# Sequencing, Analysis, and Annotation of Expressed Sequence Tags for *Camelus dromedarius*

**DOI:** 10.1371/journal.pone.0010720

**Published:** 2010-05-19

**Authors:** Abdulaziz M. Al-Swailem, Maher M. Shehata, Faisel M. Abu-Duhier, Essam J. Al-Yamani, Khalid A. Al-Busadah, Mohammed S. Al-Arawi, Ali Y. Al-Khider, Abdullah N. Al-Muhaimeed, Fahad H. Al-Qahtani, Manee M. Manee, Badr M. Al-Shomrani, Saad M. Al-Qhtani, Amer S. Al-Harthi, Kadir C. Akdemir, Mehmet S. Inan, Hasan H. Otu

**Affiliations:** 1 Biotechnology Research Center, Natural Resources and Environment Research Institute, King Abdulaziz City for Science and Technology, Riyadh, Saudi Arabia; 2 Faculty of Veterinary Medicine and Animal Resources, King Faisal University, Al-Hassa, Saudi Arabia; 3 Department of Medicine, BIDMC Genomics Center, Harvard Medical School, Boston, Massachusetts, United States of America; University of California Riverside, United States of America

## Abstract

Despite its economical, cultural, and biological importance, there has not been a large scale sequencing project to date for *Camelus dromedarius*. With the goal of sequencing complete DNA of the organism, we first established and sequenced camel EST libraries, generating 70,272 reads. Following trimming, chimera check, repeat masking, cluster and assembly, we obtained 23,602 putative gene sequences, out of which over 4,500 potentially novel or fast evolving gene sequences do not carry any homology to other available genomes. Functional annotation of sequences with similarities in nucleotide and protein databases has been obtained using Gene Ontology classification. Comparison to available full length cDNA sequences and Open Reading Frame (ORF) analysis of camel sequences that exhibit homology to known genes show more than 80% of the contigs with an ORF>300 bp and ∼40% hits extending to the start codons of full length cDNAs suggesting successful characterization of camel genes. Similarity analyses are done separately for different organisms including human, mouse, bovine, and rat. Accompanying web portal, CAGBASE (http://camel.kacst.edu.sa/), hosts a relational database containing annotated EST sequences and analysis tools with possibility to add sequences from public domain. We anticipate our results to provide a home base for genomic studies of camel and other comparative studies enabling a starting point for whole genome sequencing of the organism.

## Introduction

*Camelus dromedarius*, often referred to as the Arabian camel, is probably the most famous member of the camel family. Other members of the camel family include the llama and the alpaca in South America. The Dromedary has one hump on its back, in contrast to the Bactrian camel which has two. The Dromedary is more numerous than the Bactrian camel and represents almost 90% of the genus Camelus. Camel has historically and economically been an important species worldwide and especially in Arab Peninsula. Saudi camels comprise 16% of the animal biomass. Despite their differences, camels do have their shape in common. These differences are among breeds or within breeds. No clear classification for camels exists but generally they can be classified according to habitat, color and function. Camel breeds vary in size, body conformation and color. Color is the most common character used for classification of camel breeds. Some are dark black and others have white or brown colors. Based on their colors, three main breeds of Saudi camels were distinguished, namely black (Magaheem), white (Magateer) and brown (Al Homr and Al Sofr) [1]. Camels are multipurpose animals with females used primarily as milk producers, the males for transport or draught and both sexes providing meat as tertiary product. Camel stores its energy reserves in the form of fat in different depots in their body of which the hump and abdomen depots comprise a considerable amount of the adult body weight; therefore camels can survive long periods without feed. Hump and abdomen fats contain mixtures of fatty acids [2] and most of these are esterified as triglycerides or phospholipids and vary according to their anatomical location in the body [3]. The demand for camel meat appears to be increasing especially in arid regions. Camel meat is healthier as they produce carcasses with less fat as well as having less levels of cholesterol in fat than other meat animals [4]. Camel offers interesting biological perspectives such as heavy-chain antibody based humoral immune system [5], existence of naturally occurring domain antibodies (dAbs) (smallest known antigen-binding fragments) [6], and alternative insights in reproductive biology [7]. Moreover, camel's characteristic ability to adapt its desert lifestyle with remarkable traits such as fluctuating its body temperature from 34 degrees Celsius to 41.7 degrees Celsius throughout the day, tolerating a water loss greater than 30%, and capability of drinking 100 liters of water in as little as ten minutes [8] demands further biological investigation. Camels are usually raised in the harsh conditions of the desert, where water is scarce. These animals are well adapted to dehydration for relatively long periods [9]. Basal plasma glucose levels are significantly higher in monogastrics than in adult ruminants, but not in camels [10]. Camels are exceptional with regard to their carbohydrate metabolism. On the one hand, plasma glucose levels in camels are in the range or of those of monogastrics or even higher [11], although camels ferment carbohydrates to short-chain fatty acids on the same scale as sheep and lactating cows [12] and like ruminants, they also meet their glucose demands by endogenous gluconeogenesis [13]. On the other hand, the whole body insulin sensitivity of camels is even lower than that of adult ruminants [14]. High basal plasma glucose levels and simultaneously low insulin sensitivities also occur in humans suffering from non-insulin-dependent diabetes mellitus (NIDDM) [15], so the insulin sensitivity of camels may be similar to that of NIDDM patients.

Despite its economical, cultural, and biological importance, there has not been a large scale sequencing project targeting camel genome. We initiated Camel Genome Project with the goal of sequencing complete DNA of the organism. Since there is not much available information about the camel genome, we first established and sequenced camel EST libraries, to increase efficiency and accuracy of whole genome sequencing. A successfully completed EST library project can supply wealthy genetic information for a species, often considerably shortening the time-consuming and laborious gene isolation and characterization procedures. Large scale EST projects do not only provide direct information on the transcriptome and indirect information on correlation of genome and phenotype but also most often are very crucial to whole genome research in a given species as we have witnessed in most of mammalian genome projects. EST libraries are also very useful for molecular marker development in linkage mapping, comparative genome analysis, and estimation of gene duplication [16]. This project supplies the building backbone for comparative genomics providing a reference point from which we can compare camel with other organisms. The project helps us begin to understand the molecular basis for differences in phenotypes between camel breeds, correlation of SNPs among different camels in health and disease, disease-susceptibility prediction based on gene sequence variation, mapping and identifying genes involved in complex biological traits and multigene diseases, obtain camel specific genes to develop vaccines and/or treatments for common diseases. It can also help in the production of useful protein products for use in bioremediation and pharmaceutical industries, protein replacement (e.g. factor VIII, TPA, streptokinase, insulin, interferon), and “Pharm” animals.

In this study, we have sequenced 70,272 ESTs from three camel cDNA libraries and established a publicly available database for data mining and visualization. In order to obtain a catalogue of gene sequences found in camel irrespective of phenotype or abundance, we used normalized cDNA libraries from different age, breed, and tissue samples. Following data cleanup, clustering and assembly steps, we generated 23,602 non-redundant gene indices. We compared our findings in particular to the following nine species: *Homo sapiens*, *Mus musculus*, *Rattus norvegicus*, *Bos Taurus*, *Sus scrofa*, *Pan troglodytes*, *Macaca mulatta*, *Canis familiaris*, and *Equus caballus*. We chose human, mouse, and rat as model organisms that have been heavily worked on and have well annotated genomic data. Horse, bovine, and boar were chosen as these species are ungulates (like camel) with substantial sequence data and share many physiological properties with camel. Finally, we included monkey, chimpanzee and dog in order to lead way for comparative genomics regarding mammalian species that have found significant attention by research community in a variety of ways including sequence and genomic studies. To our knowledge data presented here represent first EST sequencing in camel and we believe accompanying web portal will become an essential tool for the annotation and assembly of camel's whole genome. Our work may have significant impact on mammalian functional genomic research since we have described over 4,500 sequences, which do not carry any homology to any other genome that is available.

## Materials and Methods

### Ethics Statement

International rules on Experimentation on Living Animals have been strictly implemented and consent has been obtained from the Saudi National Commission for Experimentation on Live Animals to carry out the relevant procedures on camels prior to the commencement of experimental work. Certification for this project has been obtained from the Saudi National Committee of Bio and Medical Ethics (Reference No: 229/131).

### Tissue Collection

Nine inbred camels of three age groups (young (0–6 months), adult (2–3 years), and aged (4–6 years)) and three different breeds (white, black, and brown coat color; determined by expert breeding techniques and visual inspection) have been used. Camels were housed in the Camel Facility at King Faisal University at Hassa, KSA. Camels were fed on alfa alfa (*Medicago sativa*) and standard concentrate diet with free access to water. All camels were humanely sacrificed by a high barbiturate dose (thiopentone sodium) for collection of tissues. We collected and pooled 11 tissues (Brain, Liver, Kidney, Heart, Muscle, Lung, Spleen, Pancreas, Stomach, Genitals, and Colon) from each of the nine camels. Pieces of dissected tissues were placed into the RNAlater solution (Ambion, USA) at room temperature. The solution permeates the cells, stabilizing the RNA. The samples were then stored at 4°C for two hours and then stored at −80°C until analyzed. In addition, blood samples were collected into Paxegen (Qiagen) tubes for RNA isolation. Since the goal of this study is not to interrogate gene expression in camel based on age, breed, tissue, or disease state but rather obtain a catalogue of gene sequences found in camel, we chose to pool different age, breed, and tissue groups to generate a collective gene expression data set in camel. This strategy attempts to capture genes expressed based on phenotypic differences. In order to obtain a most representative cDNA library for camel by trying to capture both low and high abundant transcripts expressed in different age, breed, and tissue types, we adapted a normalized cDNA library construction approach. In brief, this approach makes use of biotinylated nucleotides during *in vitro* transcription off of linearized cDNA, which are subsequently removed using streptavadin beads and phenol-chloroform, resulting in 100–200 fold reduction in highly abundant clones.

### RNA Extraction

100 mg of each tissue were homogenized in a vial containing 350 µl lyses buffer and ceramic beads using a Magnalyzer instrument (Roche Diagnostics) at 6,500 rpm. Following homogenization, the lysates were further extracted and purified by QIAamp RNA Mini Kit (Qiagen). Purified RNA samples were then analyzed by Nanodrop 1000 and Agilent 2100 Bioanalyzer RNA Nano Kit for the determination of the quantity and integrity of RNA. RNA pooling system was generated for each library. Total of 3 libraries were generated based on animal's age. Total of 2 mg of RNA for each library were used for establishing and sequencing EST libraries.

### cDNA Library Construction

First strand synthesis of the cDNA was generated by using a clamped dT primer, which is designed to attach to the poly-A junction of the mRNA. This ensures a more efficient first-strand synthesis and a shorter poly-A region at the 3′ end of the insert. cDNA fragments were size selected by running on an agarose gel. Following size selection, cDNA ends are polished and the cDNAs were digested using a rare cutting enzyme and were directionally cloned into pAGEN-1 vector.

Single stranded DNA was made from a portion of the primary library by phagemid production and to ensure elimination of the double stranded DNA contamination from the single stranded DNA preb; the reactions were then digested with DNAse I. A second portion of the primary library was linearized and transcribed into anti-sense RNA with biotinylated dNTPs. oligo dT and primer extension were used to pre-block the poly-A region prior to hybridization. This prevents hybridization of the poly-A clone and the poly-U of the RNA. The anti-sense RNA and single stranded circular DNA were then hybridized and abundant clones were removed using streptavidin. To prevent any empty vectors, A *Not1* oligo and *Taq* polymerase were used to synthesize double stranded DNA from the single stranded normalized library.

### EST Data Analysis

**Figure 1:**
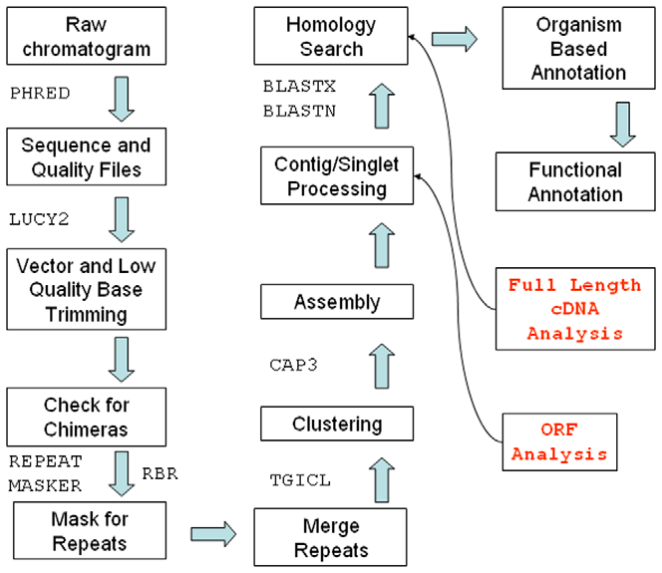
Analysis Workflow: Outlay of analysis steps performed for Camel EST data. External programs used for analysis are shown where appropriate.

Using raw chromatogram files, we obtained corresponding sequence and quality values, which results in base calls and associated scores regarding the accuracy of the call for each EST sequence, or “read” in the libraries. Reads thus obtained were analyzed for vector sequence contamination and removal of low quality bases. The remaining high quality reads were checked for the existence of chimeric sequences. Upon fixing for chimeras, reads were masked for known and estimated repeats. These masked regions were not used for similarity calculation required in the clustering step, which groups reads showing high sequence similarity. Contigs and singletons obtained from alignment of reads in each cluster imply putative gene sequences, which were analyzed for homology searches in existing sequence databases and open reading frame (ORF) discovery. The results were annotated using Gene Ontology functional classification for various species that the putative gene sequences have been matched to. An overall summary of the analysis pipeline is depicted in Figure 1.

### Quality Calls and Trimming

We started with 23,424 chromatogram files from each library, equaling 70,272 reads overall. Raw chromatogram files were processed using PHRED, which reads trace data, calls bases, and assigns quality values to bases using Fourier transforms [17], [18]. We removed low quality regions, vector contamination and potential polyA regions using Lucy2 [19], determining the longest continuous high quality region for each read. If the remaining sequence after the clean-up procedure was less than 100 base pairs (bp), we eliminated the sequence from downstream analysis.

### Identification of Chimeric Sequences

During cloning process, two or more DNA fragments can be ligated together yielding a chimeric EST read. Chimeras are usually PCR artifacts thought to occur when a prematurely terminated amplicon reanneals to a foreign DNA strand and is copied to completion in the following PCR cycles. We identified chimeric sequences by searching for internally inserted adaptors or cloning sites in the reads [20]. When such a sequence is found, we calculated the lengths of the subsequences after the chimeric sequence is divided into two or more pieces. If the length of any such subsequence was less than 50 bp, the subsequence was removed from downstream analysis. We performed chimera analysis on the reads trimmed of low quality regions and vector sequences.

### Masking Repeats

Repeat regions could result in artificial sequence similarity by being used as “seeds” in alignment process [21]. In order to prevent this, we adapted two strategies to mask repeats. The first is the widely used RepeatMasker algorithm that uses a library of known repeats [22]. For this purpose, we used “*Camelus dromedarius*” as the reference organism in the RepeatMasker algorithm. However, since the whole genome of the Arabian camel is not available, the algorithm uses a library that contains the first common ancestor of *Camelus dromedarius* for which a repeat library has been constructed.

For this very reason that we lack a whole genome for camel, we adopted a second approach to mask repeats [23]. In this “libraryless” repeat masking procedure, RBR, we first identified words (a small stretch of DNA sequence) of length at least 16 bp in all EST sequences. Next, a distribution for the frequency of word repeats is obtained. If a word's frequency was above 5 standard deviation of the expected number of repeats for a word, we concluded that such a repeat could not have been caused by resampling of the same mRNA. Therefore such words with high occurrences are regarded to be represented in the EST data set due to DNA repeats. We masked a base pair if it was called to be in a repeat region by either of the two algorithms.

### Clustering and Assembly

We clustered reads using The Gene Indices Clustering Tool (TGICL) [24] and for each cluster performed multiple alignment of its members using CAP3 (Huang and Madan, 1999). The consensus sequence thus obtained for each cluster approximates the target mRNA sequence represented by the reads in the cluster. When performing assembly for a group of reads in a given cluster, reads that do not fit well in the overall alignment are dropped out and not included in the final formation of the “contigs”, the consensus sequence representing a cluster. Such dropped out reads combined with reads that are placed in self-contained clusters are referred to as “singletons”.

### Homology Search and Functional Annotation

We calculated the longest ORF for each contig and singleton sequence using NCBI's standard genetic code to relate homology searches to the availability of ORFs in the input set. We then compared each contig and singleton to NCBI's nucleotide (nt; containing 6,944,581 sequences) and non-redundant protein (nr; containing 6,655,203 sequences) databases using BLASTN (E-value cut-off 10^−30^) and BLASTX (E-value cut-off 10^−5^) algorithms, respectively [25]. Resulting “hits” were analyzed separately for nine species. We also searched for hits in the NIH Mammalian Gene Collection Project (http://mgc.nci.nih.gov) to identify matches to full-length cDNA sequences for the following species: *Homo sapiens*, *Mus musculus*, *Rattus norvegicus*, and *Bos Taurus* [26]. This search was conducted using BLASTX and results were analyzed to identify coverage of match regions over start and/or stop codons of full-length cDNA sequences. A hit was identified if the alignment between query and hit sequences was over 30 amino acids (aa) with at least 96% identity.

Functional annotation of reads that are mapped to homologous sequences using BLAST is done by identifying the Gene Ontology (GO) categories for the hits [27]. We identified Biological Process, Cellular Component, and Molecular Function categories seen in our matched sequences using NCBI's GENE database mapping to GO terms. Our sequence and functional annotation strategy was NCBI's gi number centric: for every contig or singleton, the sequence's hits' gi numbers were pooled and matched to unique GeneIDs to remove redundancy. Hence match results presented always refer to unique number of genes found in NCBI's GENE database. These GeneIDs were then mapped to GO terms.

In an attempt to perform comparative genomic analysis, we compared genes mapped from our EST database to human, mouse, rat, and bovine using NCBI's HomoloGene database. We found genes shared by all four organisms and analyzed them with Ingenuity Pathway Analysis (IPA) software (Ingenuity® Systems, www.ingenuity.com). IPA generates networks based on the connectivity of genes in the data set using Ingenuity Knowledge Base, which is mostly dependent on scientific literature. IPA performs functional analysis by identifying biological functions and/or diseases that are most significant to the data set using right tailed Fisher's exact test. This functional analysis is similarly applied to molecules in a given network generated by IPA for the data set. IPA also projects molecules in the data set to known biological pathways (referred to as “canonical pathways”) and finds significantly associated canonical pathways by calculating the ratio of molecules in the data set mapped to canonical pathway divided by total number of molecules in the canonical pathway and by using Fisher's exact test to assign significance to observed association.

## Results

**Figure 2:**
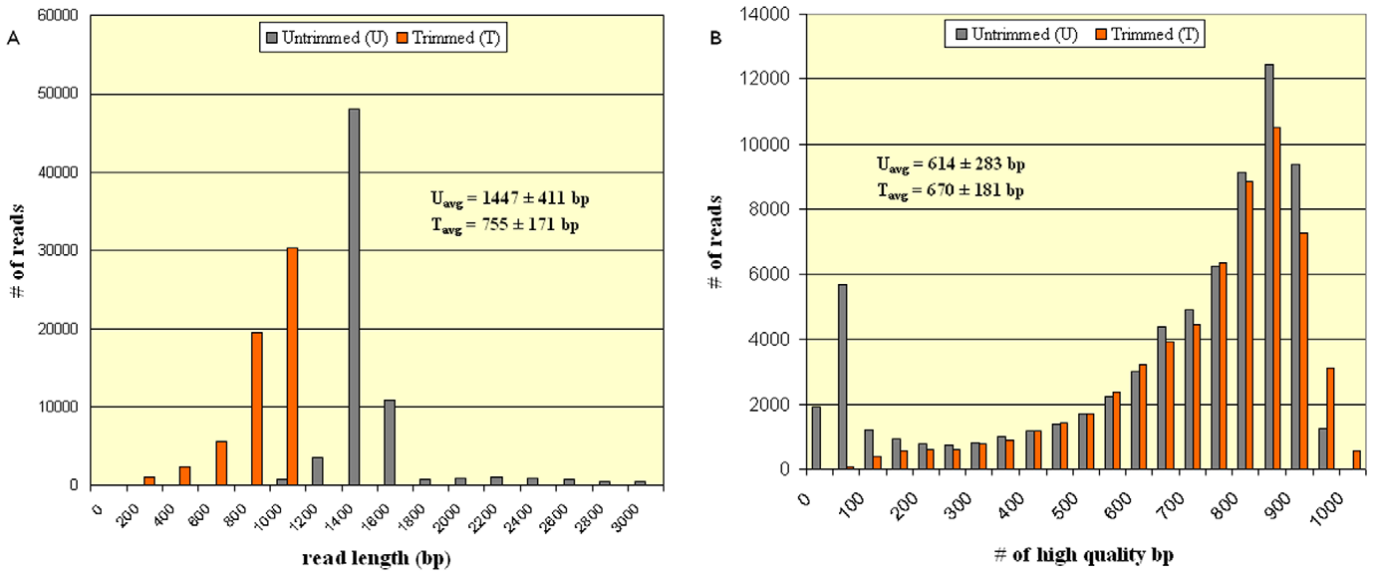
Read Length and Base Call Quality Distribution: Distribution of read length (a) and high quality base pairs per read (b) in trimmed and untrimmed EST data. Average ± standard deviation values for the measured parameters are overlaid on the graphs.

cDNA library created from 11 tissues of three different inbred camels of different ages produced enough material to sequence around 80,000 clones. 23,424 EST sequences were determined from each library by single pass 5′ sequencing yielding a total of 70,272 EST sequences, with an average read length of 1447±411 (avg. ± st.dev.) bp and an average high quality (Phrap score >20) per read of 614±283 bp. After trimming vector contamination, low quality bases, and eliminating trimmed sequences with length less than 100 bp, three libraries resulted in 58,842 high-quality EST sequences with an average read length of 755±171 bp and an average high quality base pairs per read of 670±181. These results are summarized in Table 1. The increase of average high quality base pairs per read from 614 (untrimmed) to 670 (trimmed) indicates successful elimination of reads with a high abundance of low quality bases. Moreover, the ratio of average high quality base pairs per read to average read length increased from 42% to 88% after trimming. Tightness in the variation of read length and number of high quality bases per read following trimming indicates a high quality set of reads for downstream analysis (see Figure 2).

**Table 1:** Summary of EST analysis results.

Read Statistics		Sequence Statistics	
Untrimmed # of reads	70,272	# of contigs/singletons	8,319/15,283
Average read length	1,447±411 bp	average # of reads per contig	5.2
Average # of high quality bp/read	614±283	average contig length	1,247 bp
# of reads after trimming	58,842	average singleton length	696 bp
Average read length	755±171 bp	average ORF length (contig)	673 bp
Average # of high quality bp/read	670±181	average ORF length (singleton)	390 bp
# of chimeric sequences	1,241	# of contigs with hit	7,490
# of reads after chimera analysis	59,534	# of singletons with hit	11,480
# of reads with repeat region	18,340 (30.8%)	# of contigs with no hit	829
total # of bp masked due to repeats	∼2.5×10^6^ (5.5%)	# of singletons with no hit	3,803

We identified 1,241 chimeric sequences, accounting for about 2% of high-quality reads. After splitting chimeric sequences and removing remaining subsequences that are less than 50 bp, we were left with 59,534 reads, an increase of about 1% in number of reads. RepeatMasker and RBR found repeats in about 10% and 20% of the reads, respectively, though RepeatMasker masked ∼1.5×10^6^ bp, roughly 1.5 times more bp than masked by RBR. When we combined regions masked by either method, about 30% of all the reads and roughly ∼2.5×10^6^ bp were masked suggesting both sequences and regions masked by the two methods were almost exclusive. These results imply that our approach to repeat finding identifies regions that could have potentially been missed if either of the programs were solely used; an approach adapted by most large-scale sequencing projects.

**Figure 3:**
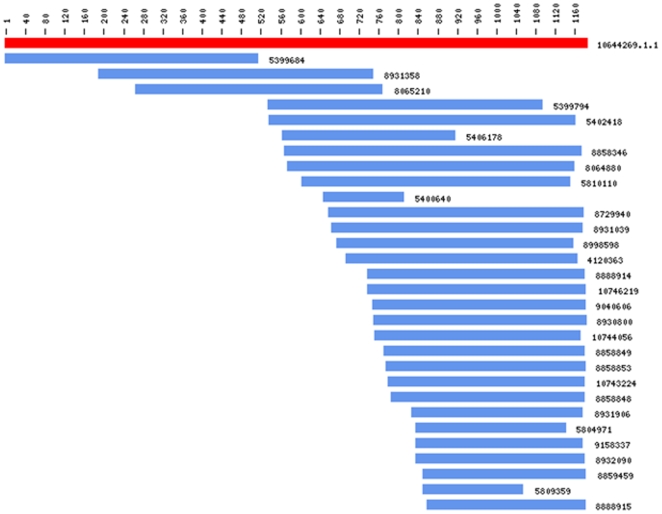
Sample Cluster: A sample instance of a cluster showing thirty high quality reads masked for repeats that are grouped and aligned to form a final consensus sequence yielding a contig. Individual reads are shown as blue bars and the consensus sequence is shown at top as a red bar. Labels to the left of bars show sequence IDs used for internal analysis purposes. Base pair scale is shown above the consensus sequence with 40 bp intervals rendering a consensus sequence slightly above 1,160 bp. Reads render overlaps of at least 98% identity, at least 40bp, and at most 20bp overlap distance of sequence end.

**Figure 4:**
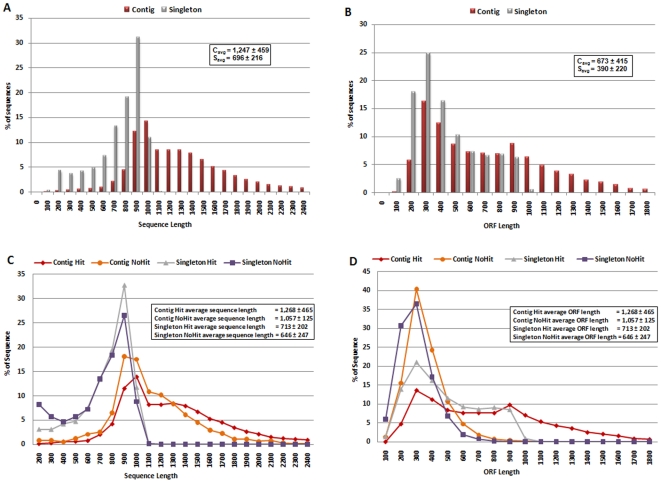
Sequence Length and ORF Length Distribution: Sequence length distribution for contigs and singletons (a), distribution of longest ORF lengths found in contigs and singletons (b), sequence length distribution for contigs and singletons with hits and no hits (c), and distribution of longest ORF lengths found in contigs and singletons with hits and no hits (d). Average ± standard dev. values of sequence and ORF lengths are overlaid on corresponding graphs. Sequence lengths up to 2,500 and ORF lengths up to 1,800 bp are shown for display purposes. 2.3% of contig and no singleton sequences have length longer than 2,500 bp (a), 2% of contig and no singleton sequences have an ORF longer than 1,800 bp (b), 1.8% of contigs with a hit and no other sequences in the remaining three groups have length longer than 2,500 bp (c), and 1.6% of contigs with a hit and no other sequences in the remaining three groups have an ORF longer than 1,800 bp (d).

High-quality EST sequences masked for repeats were assembled by TGICL resulting in 8,319 contigs and 15,283 singletons yielding 23,602 putative gene sequences. In Figure 3, we show an instance of a cluster and assembled consensus sequence containing thirty reads. In forming this contig, we considered reads with overlaps of at least 98% identity, at least 40bp, and at most 20bp overlap distance of sequence end. Overall, average number of reads in a cluster was 5.2 (see Table 1). Average sequence length for the contigs and singletons were 1,247±459 bp and 696±216 bp (avg. ± st.dev.), respectively (see Table 1). In Figure 4a, we show sequence length distribution where sequences of length longer than 2,500 are not shown for display purposes. About 2.3% of contig and no singleton sequences have length longer than 2,500 bp. This difference in sequence length is expected as contigs are supported by multiple reads. On the other hand, sequence lengths are long enough to suggest identification of full or partial coding sequences. In Figure 4b, we show length distribution of the longest ORF found in contigs and singletons. As expected, contigs yield longer ORFs (avg. length: 673±415 bp) with ∼80% of contigs having an ORF longer than 300 bp suggesting successful characterization of gene coding regions found in camel DNA. ∼55% of singletons have an ORF longer than 300 bp (avg. ORF length: 390±220 bp; see Table 1). This difference is expected as the starting average length for contigs are higher than that of singletons. About 20% of the contigs have an ORF longer than 1,000 bp; 2% of which are longer than 1,800 bp, not shown in Figure 4b for display purposes.

In order to identify likely camel genes through sequence homology, BLAST analysis was performed on 23,602 contigs and singletons against NCBI's nucleotide and non-redundant protein databases. Out of 8,319 contigs 7,490 (90%) sequences got a hit and 829 (10%) sequences got no hit. Similarly, for 15,283 singletons, there were 11,480 (75%) and 3,803 (25%) sequences with a hit and no hit, respectively. Overall, 18,970 (80%) sequences were hit with one or more matches and there were 4,632 (20%) sequences that showed no significant similarity to any of the sequences in the databases. 4,632 sequences with “no hits” may represent novel camel specific genes or genes that evolve relatively quickly.

We also analyzed length and ORF distributions in contigs and singletons based on their “hit” status. These results are shown in Figure 4c,d. The average length ± st. dev. for contig sequences that got hit and no hit were 1,268±465 bp and 1,057±125 bp, respectively. Similarly, average length ± st. dev. values were 713±202 bp and 646±247 bp for singleton sequences that got hit and no hit, respectively. These results show about 16.6% and 9.4% decrease in average lengths between sequences with a hit and no hit for contig and singleton sequences, respectively. As the length of reads that got no hit are not dramatically smaller, we believe this indicates potential discovery of novel coding regions in camel. On the other hand, the average length ± st. dev. of longest ORFs found in contig sequences that got hit and no hit were 714±416 bp and 306±125 bp, respectively. The difference in average ORF length between contigs that got hit and no hit is about 57%, significantly larger than the average length difference for the two groups. We observe a similar behavior for singletons where average length ± st. dev. values were 437±227 bp and 248±112 bp (∼43% difference) for longest ORF lengths found in singleton sequences that got hit and no hit, respectively. Although reads that got no hit show small ORF lengths, there were 362 contig and 1,409 singleton “no hit” sequences with an ORF longer than 300 bp suggesting ∼40% of potentially novel genes show strong coding capacity.

We further analyzed BLAST results separately for nine species. These results are summarized in Table 2. For example, 85% of all contigs found a hit in human and the average ORF length of these contigs were 740 bp. 84% of the contigs that found a match in human contained an ORF longer than 300 bp. Overall, we generally have higher 70s to 80% of contigs and 50–60% of singletons with a hit in the nine species when analyzed separately. Moreover, the average ORF lengths were 700–800 bp for contigs and 400–500 bp for singletons with about 90% of contigs and 60–70% of singletons having an ORF length greater than 300 bp. These results suggest that when reads with a hit are analyzed in a specific species, we most likely find homologous gene sequences with coding capacity. We also found genes that got most hits by camel sequences for the nine species. A complete list for each analyzed species can be found in CAGBASE and lists of top fifty genes for human, mouse, rat, and bovine are shown in Table 3, Table 4, Table 5 and Table 6. We also compared our EST sequences to all 674 available *Camelus Dromedarius* mRNA sequences in NCBI GenBank. Out of these 674 sequences only 16 were not matched significantly. The results for all 674 sequences along with top 10 hits from our camel EST database can be found in CAGBASE.

**Table 2:** BLAST results for contigs, singletons, and their combination shown separately for the nine species analyzed.

Species	Contigs			Singletons			Combined		
	Sequences with a hit	Avg. ORF Length	Sequences with ORF>300 bp	Sequences with a hit	Avg. ORF Length	Sequences with ORF>300 bp	Sequences with a hit	Avg. ORF Length	Sequences with ORF>300 bp
*Homo s.*	7,045 (85%)	740	5,938 (84%)	10,247 (67%)	458	7,047 (69%)	17,292 (73%)	573	12,985 (75%)
*Mus m.*	6,323 (76%)	783	5,597 (89%)	8,373 (55%)	467	6,064 (72%)	14,696 (62%)	603	11,661 (78%)
*Rattus n.*	6,032 (73%)	803	5,455 (90%)	7,824 (51%)	479	5,873 (75%)	13,856 (59%)	620	11,328 (82%)
*Bos t.*	6,440 (77%)	775	5,656 (88%)	8,681 (57%)	469	6,240 (72%)	15,121 (64%)	599	11,896 (79%)
*Equus c.*	5,737 (69%)	811	5,195 (91%)	7,590 (50%)	487	5,699 (75%)	13,327 (57%)	626	10,894 (82%)
*Canis f.*	5,965 (72%)	802	5,367 (90%)	7,778 (51%)	469	5,707 (73%)	13,743 (58%)	614	11,074 (81%)
*Macaca m.*	6,462 (78%)	772	5,640 (87%)	8,586 (56%)	481	6,337 (74%)	15,048 (64%)	606	11,977 (80%)
*Pan t.*	6,516 (78%)	766	5,645 (87%)	8,530 (56%)	472	6,181 (72%)	15,046 (64%)	599	11,826 (79%)
*Sus s.*	1,831 (22%)	846	1,684 (92%)	2,289 (15%)	515	1,764 (77%)	4,120 (17%)	662	3,448 (84%)

Percentage of sequences that got a hit to the total number of sequences in each group (contig, singleton, or combined) is shown separately for each species. For the sequences that got a hit, average ORF length and the percentage of sequences with ORF >300 bp (to the total number of sequences that got a hit) is shown for each group and species.

**Table 3:** Most frequently matched genes in human.

Rank	GeneID	Gene_Symbol	Gene Name (*Homo sapiens*)
1	3507	IGHM	immunoglobulin heavy constant mu
2	3500	IGHG1	immunoglobulin heavy constant gamma 1 (G1m marker)
3	28396	IGHV4-31	immunoglobulin heavy variable 4-31
4	3492	IGH@	immunoglobulin heavy locus
5	3502	IGHG3	immunoglobulin heavy constant gamma 3 (G3m marker)
6	3501	IGHG2	immunoglobulin heavy constant gamma 2 (G2m marker)
7	3503	IGHG4	immunoglobulin heavy constant gamma 4 (G4m marker)
8	100133739	LOC100133739	similar to hCG2038920
9	3493	IGHA1	immunoglobulin heavy constant alpha 1
10	652494	LOC652494	similar to Ig heavy chain V-III region VH26 precursor
11	3495	IGHD	immunoglobulin heavy constant delta
12	100126583	LOC100126583	hypothetical LOC100126583
13	80314	EPC1	enhancer of polycomb homolog 1 (Drosophila)
14	28412	IGHV3-66	immunoglobulin heavy variable 3-66
15	3105	HLA-A	major histocompatibility complex, class I, A
16	3107	HLA-C	major histocompatibility complex, class I, C
17	717	C2	complement component 2
18	28393	IGHV4-55	immunoglobulin heavy variable 4-55
19	28417	IGHV3-60	immunoglobulin heavy variable 3-60
20	28464	IGHV1-58	immunoglobulin heavy variable 1-58
21	28392	IGHV4-59	immunoglobulin heavy variable 4-59
22	28418	IGHV3-57	immunoglobulin heavy variable 3-57
23	28380	IGHV7-56	immunoglobulin heavy variable 7-56
24	28419	IGHV3-54	immunoglobulin heavy variable 3-54
25	643406	LOC643406	hypothetical protein LOC643406
26	28424	IGHV3-48	immunoglobulin heavy variable 3-48
27	4276	MICA	MHC class I polypeptide-related sequence A
28	8449	DHX16	DEAH (Asp-Glu-Ala-His) box polypeptide 16
29	440716	LOC440716	hypothetical LOC440716
30	203068	TUBB	tubulin, beta
31	10919	EHMT2	euchromatic histone-lysine N-methyltransferase 2
32	1192	CLIC1	chloride intracellular channel 1
33	3304	HSPA1B	heat shock 70kDa protein 1B
34	3303	HSPA1A	heat shock 70kDa protein 1A
35	7919	BAT1	HLA-B associated transcript 1
36	23	ABCF1	ATP-binding cassette, sub-family F (GCN20), member 1
37	3135	HLA-G	major histocompatibility complex, class I, G
38	10107	TRIM10	tripartite motif-containing 10
39	2794	GNL1	guanine nucleotide binding protein-like 1
40	7726	TRIM26	tripartite motif-containing 26
41	10255	HCG9	HLA complex group 9
42	80739	C6orf25	chromosome 6 open reading frame 25
43	259197	NCR3	natural cytotoxicity triggering receptor 3
44	7918	BAT4	HLA-B associated transcript 4
45	89870	TRIM15	tripartite motif-containing 15
46	80740	LY6G6C	lymphocyte antigen 6 complex, locus G6C
47	55937	APOM	apolipoprotein M
48	58530	LY6G6D	lymphocyte antigen 6 complex, locus G6D
49	1460	CSNK2B	casein kinase 2, beta polypeptide
50	7124	TNF	tumor necrosis factor (TNF superfamily, member 2)

**Table 4:** Most frequently matched genes in mouse.

Rank	GeneID	Gene_Symbol	Gene Name (*Mus musculus*)
1	111507	Igh	immunoglobulin heavy chain complex
2	380794	Ighg	Immunoglobulin heavy chain (gamma polypeptide)
3	100043989	LOC100043989	V(H)76 segment leader peptide
4	100047678	LOC100047678	similar to pORF2
5	380795	AI324046	expressed sequence AI324046
6	16019	Igh-6	immunoglobulin heavy chain 6 (heavy chain of IgM)
7	195176	Igh-VX24	immunoglobulin heavy chain (X24 family)
8	790956	LOC790956	5.8S ribosomal RNA
9	19791	Rn18s	18S RNA
10	236598	LOC236598	28S ribosomal RNA
11	22138	Ttn	Titin
12	100045801	LOC100045801	similar to pORF2
13	320473	Heatr5b	HEAT repeat containing 5B
14	234358	D10627	cDNA sequence D10627
15	100042306	100042306	predicted gene, 100042306
16	56314	Zfp113	zinc finger protein 113
17	233058	Zfp420	zinc finger protein 420
18	100041343	100041343	predicted gene, 100041343
19	234542	Rtbdn	Retbindin
20	22678	Zfp2	zinc finger protein 2
21	16061	Igh-VJ558	immunoglobulin heavy chain (J558 family)
22	16059	Igh-V7183	immunoglobulin heavy chain (V7183 family)
23	100040270	OTTMUSG00000013146	predicted gene, OTTMUSG00000013146
24	76958	2210418O10Rik	RIKEN cDNA 2210418O10 gene
25	100039123	OTTMUSG00000016219	predicted gene, OTTMUSG00000016219
26	12937	Pcdha6	protocadherin alpha 6
27	244556	Zfp791	zinc finger protein 791
28	170833	Hook2	hook homolog 2 (Drosophila)
29	21672	Prdx2	peroxiredoxin 2
30	22704	Zfp46	zinc finger protein 46
31	16477	Junb	Jun-B oncogene
32	436049	EG436049	predicted gene, EG436049
33	246196	Zfp277	zinc finger protein 277
34	624855	EG624855	predicted gene, EG624855
35	19283	Ptprz1	protein tyrosine phosphatase, receptor type Z, polypeptide 1
36	68628	Fbxw9	F-box and WD-40 domain protein 9
37	17159	Man2b1	mannosidase 2, alpha B1
38	414077	BC056474	cDNA sequence BC056474
39	68544	2310036O22Rik	RIKEN cDNA 2310036O22 gene
40	56495	Asna1	arsA (bacterial) arsenite transporter, ATP-binding, homolog 1
41	67836	1500041N16Rik	RIKEN cDNA 1500041N16 gene
42	212989	Best2	bestrophin 2
43	212999	Tnpo2	transportin 2 (importin 3, karyopherin beta 2b)
44	380800	Ighvq52.3.8	immunoglobulin heavy chain variable region Q52.3.8
45	69724	Rnaseh2a	ribonuclease H2, large subunit
46	330817	Dhps	deoxyhypusine synthase
47	22259	Nr1h3	nuclear receptor subfamily 1, group H, member 3
48	19732	Rgl2	ral guanine nucleotide dissociation stimulator-like 2
49	100039000	100039000	predicted gene, 100039000
50	16971	Lrp1	low density lipoprotein receptor-related protein 1

**Table 5:** Most frequently matched genes in rat.

Rank	GeneID	Gene_Symbol	Gene Name (*Rattus norvegicus*)
1	299354	Ighg	Immunoglobulin heavy chain (gamma polypeptide)
2	367586	IgG-2a	gamma-2a immunoglobulin heavy chain
3	498354	LOC498354	hypothetical protein LOC498354
4	361915	LOC361915	hypothetical protein LOC361915
5	294421	Serinc1	serine incorporator 1
6	498550	RGD1560705	similar to LRRGT00152
7	362795	LOC362795	immunoglobulin G heavy chain
8	25419	Crp	C-reactive protein, pentraxin-related
9	299352	Igh-1a	immunoglobulin heavy chain 1a (serum IgG2a)
10	501173	LOC501173	hypothetical protein LOC501173
11	309243	Vps13a	vacuolar protein sorting 13A (yeast)
12	317588	LOC317588	hypothetical protein LOC317588
13	361942	LOC361942	similar to ORF4
14	681893	LOC681893	similar to SET protein
15	501553	LOC501553	hypothetical protein LOC501553
16	25116	Hsd11b1	hydroxysteroid 11-beta dehydrogenase 1
17	299357	RGD1359202	similar to immunoglobulin heavy chain 6 (Igh-6)
18	366747	LOC366747	similar to Ig heavy chain V region MC101 precursor
19	314509	LOC314509	similar to single chain Fv antibody fragment scFv 7–10A
20	499136	LOC499136	LRRGT00021
21	299458	LOC299458	similar to Ig H-chain V-region precursor
22	499120	LOC499120	hypothetical protein LOC499120
23	314487	Igha_mapped	immunoglobulin heavy chain (alpha polypeptide) (mapped)
24	24233	C4a	complement component 4a
25	24231	C2	complement component 2
26	361798	Ehmt2	euchromatic histone lysine N-methyltransferase 2
27	294257	Cfb	complement factor B
28	497897	Zfp2	zinc finger protein 2
29	406864	Clic1	chloride intracellular channel 1
30	294254	Hspa1b	heat shock 70kD protein 1B (mapped)
31	55939	Apom	apolipoprotein M
32	294260	Skiv2l	superkiller viralicidic activity 2-like
33	24472	Hspa1a	heat shock 70kD protein 1A
34	24591	Neu1	neuraminidase 1
35	25009	Vars2	valyl-tRNA synthetase 2
36	294255	Slc44a4	solute carrier family 44, member 4
37	406171	G7e	G7e pseudogene
38	309613	Ng35	Ng35 pseudogene
39	309609	Ly6g6f	lymphocyte antigen 6 complex, locus G6F
40	406866	Ly6g6e	lymphocyte antigen 6 complex, locus G6E
41	361799	Dom3z	DOM-3 homolog Z (C. elegans)
42	361796	Bat5	HLA-B associated transcript 5
43	81650	Csnk2b	casein kinase 2, beta subunit
44	309611	G7c	G7c protein
45	361800	Stk19	serine/threonine kinase 19
46	294241	Ly6g6c	lymphocyte antigen 6 complex, locus G6C
47	415064	Bat4	Bat4 gene
48	406170	Ng23	Ng23 protein
49	415062	Ly6g6d	lymphocyte antigen 6 complex, locus G6D
50	94342	Bat3	HLA-B-associated transcript 3

**Table 6:** Most frequently matched genes in bovine.

Rank	GeneID	Gene_Symbol	Gene Name (*Bos taurus*)
1	281850	IGHG1	immunoglobulin heavy constant gamma 1
2	281852	IGHG3	immunoglobulin heavy constant gamma 3
3	404060	IGG1C	IgG1 heavy chain constant region
4	790411	LOC790411	endonuclease reverse transcriptase
5	503551	BTIGGHB	C-H-gamma pseudogene, psi-gamma
6	508062	ZNF135	zinc finger protein 135
7	522642	LOC522642	similar to Zinc finger protein 420
8	767896	ZFP2	zinc finger protein 2 homolog
9	519934	H2B	histone H2B-like
10	504943	RXRB	retinoid X receptor, beta
11	282492	BOLA-DNA	major histocompatibility complex, class II, DN alpha
12	282497	BOLA-DYA	major histocompatibility complex, class II, DY alpha
13	515435	COL11A2	collagen, type XI, alpha 2
14	524959	TAP1	transporter 1, ATP-binding cassette, sub-family B (MDR/TAP)
15	282013	PSMB8	proteasome (prosome, macropain) subunit, beta type, 8
16	512468	GCLC	glutamate-cysteine ligase, catalytic subunit
17	510593	PSMB9	proteasome (prosome, macropain) subunit, beta type, 9
18	505358	BRD2	bromodomain containing 2
19	532422	HSD17B8	hydroxysteroid (17-beta) dehydrogenase 8
20	282490	BOLA-DMA	major histocompatibility complex, class II, DM alpha-chain, expressed
21	282493	BOLA-DOB	major histocompatibility complex, class II, DO beta
22	282491	BOLA-DMB	major histocompatibility complex, class II, DM beta-chain, expressed
23	282498	BOLA-DYB	major histocompatibility complex, class II, DY beta
24	540716	SLC39A7	solute carrier family 39 (zinc transporter), member 7
25	618722	LOC618722	similar to MHC class II antigen
26	618733	TAP2	transporter 2, ATP-binding cassette, sub-family B (MDR/TAP)
27	614564	ZNF79	zinc finger protein 79
28	512364	ZNF84	zinc finger protein 84
29	100124518	LOC100124518	hypothetical protein LOC100124518
30	404057	IGHM	immunoglobulin heavy constant mu
31	514023	ZNF180	zinc finger protein 180
32	522837	LOC522837	hypothetical LOC522837
33	618141	ZNF3	zinc finger protein 3
34	524256	ZNF300	zinc finger protein 300
35	783710	LOC783710	similar to ENSANGP00000009498
36	515674	ZNF184	zinc finger protein 184
37	520008	ZNF569	zinc finger protein 569
38	513814	ZNF16	zinc finger protein 16
39	506448	ZNF397	zinc finger protein 397
40	511931	LOC511931	hypothetical LOC511931
41	539552	ZNF167	zinc finger protein 167
42	510417	BOLA-NC1	non-classical MHC class I antigen
43	530050	MYH11	myosin, heavy chain 11, smooth muscle
44	786931	LOC786931	similar to Zinc finger protein 585A
45	518207	ZNF345	zinc finger protein 345
46	493779	LOC493779	18S ribosomal RNA
47	508355	ITIH3	inter-alpha (globulin) inhibitor H3
48	505478	IGL@	immunoglobulin light chain, lambda gene cluster
49	539265	ZNF502	zinc finger protein 502
50	515712	BOLA	MHC class I heavy chain

**Figure 5:**
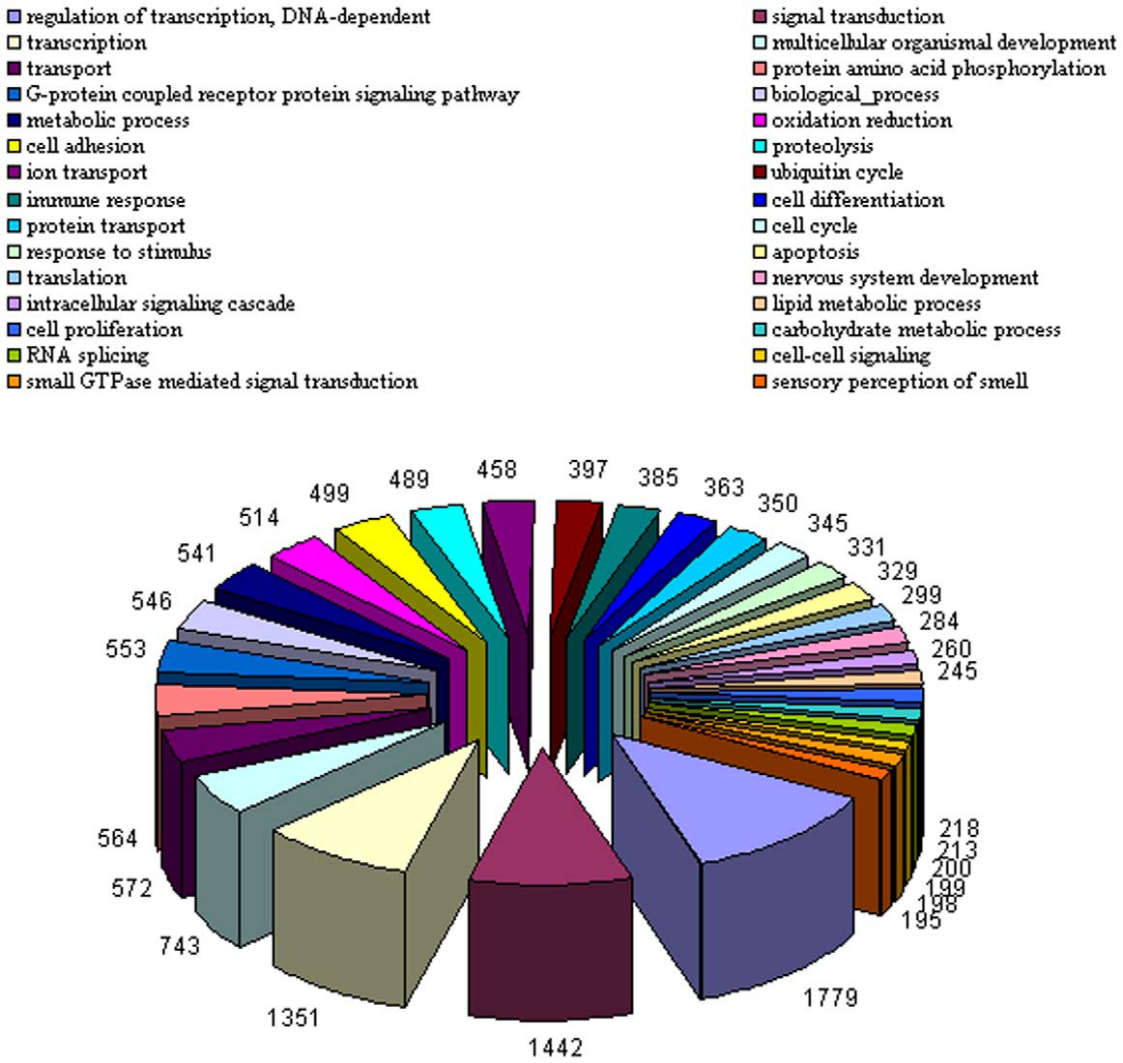
GO Categories: Top thirty Biological Process GO category terms found most abundant among the *Homo sapiens* genes similar to camel sequences.

Table 7 summarizes functional annotation results for putative camel gene sequences. Overall, regarding species for which GO annotation is available, around 70% of genes that got a hit contain a GO term, corresponding to about 90% of all the GO terms found in the organism. For example, when all camel sequences are considered in combination, compared to *Mus musculus*, 14,390 GeneIDs (or 65%) out of 22,212 GeneIDs that are hit contain a GO term amounting to 6,098 distinct GO terms, which is 95% of a total of 6,452 distinct GO terms found in the species. These results suggest that identified camel sequences capture most of functional characteristics described in other species. In Figure 5, we show top highly abundant Biological Process GO Terms found in camel among the genes mapped to *Homo sapiens*. When most frequently matched functional GO categories are studied, we see a broad range of functional terms but interestingly some that could relate to properties more commonly attributable to camel such as “oxidation reduction” and “sensory perception of smell”. A complete list of GO results for all four species can be found in online CAGBASE web portal.

**Table 7:** Numbers of unique GeneIDs, GO Terms that are mapped by the ESTs with hits for the nine species analyzed.

Species				Contigs			Singletons			Combined		
	Gene IDs	GO Terms	Gene IDs with GO Term	Gene IDs Hit	GO Terms Mapped	Hit Gene IDs with GO	Gene IDs Hit	GO Terms Mapped	Hit Gene IDs with GO	Gene IDs Hit	GO Terms Mapped	Hit Gene IDs with GO
*Homo s.*	39,920	6,897	18,370	19,113	6,296	13,673	19,799	6,410	14,206	22,980	6,664	15,934
*Mus m.*	63,648	6,452	18,047	18,109	5,724	12,210	17,584	5,762	12,105	22,212	6,098	14,390
*Rattus n.*	37,838	7,256	13,330	10,805	5,709	6,803	11,018	5,870	6,926	14,991	6,520	9,003
*Bos t.*	29,496	3,780	11,972	10,459	2,851	5,948	11,019	2,850	6,046	14,766	3,290	7,984
*Equus c.*	23,876	N/A	N/A	7,760	N/A	N/A	8,249	N/A	N/A	10,868	N/A	N/A
*Canis f.*	20,187	N/A	N/A	9,267	N/A	N/A	9,776	N/A	N/A	12,941	N/A	N/A
*Macaca m.*	29,187	N/A	N/A	11,174	N/A	N/A	10,935	N/A	N/A	15,512	N/A	N/A
*Pan t.*	31,555	N/A	N/A	11,059	N/A	N/A	10,738	N/A	N/A	15,254	N/A	N/A
*Sus s.*	3,506	N/A	N/A	1,437	N/A	N/A	1,360	N/A	N/A	1,800	N/A	N/A

Numbers of GeneIDs, GO Terms, and GeneIDs that have a GO annotation are shown for the nine species analyzed, where applicable. For each camel sequence group (contig, singleton, and combination of the two), number of unique GeneIDs that are “hit” by BLAST analyses are shown. Where applicable, we also show number of GO terms mapped by the GeneIDs that got hit and number GeneIDs among this list that have a mapped GO term.

We compared camel ESTs to full-length cDNA sequences of *Homo sapiens*, *Mus musculus*, *Rattus norvegicus*, and *Bos Taurus* containing 28,133; 23,120; 5,341; and 9,188 sequences, respectively. Summarized in Table 8, we identified 2,089; 1,543; 884; and 1,646 camel ESTs that show high similarity (96% identity over at least 30 aa) to full-length cDNA sequences of aforementioned organisms, respectively. In total, we have 2637 camel ESTs that matched to a full-length cDNA in any of the four organisms, corresponding to about 11% of all camel sequences. A complete list of full length cDNA analysis is hosted at CAGBASE web portal. We found that in 35–50% of matching contigs, the alignment was within 5 aa of the start codon, suggesting that near-complete coding sequences have been obtained for these genes. Lack of a similar database for camel unfortunately prevents these results to be processed to better estimate sequences representing full length cDNAs and total number of genes in camel.

**Table 8:** Number of Camel sequences that showed high similarity (>96% identity over at least 30 aa) to known full length cDNA sequences.

Organism	# of full length cDNA	matched (contig)	matched (singleton)	matched (combined)	N-terminus proximal
*Bos t.*	9,188	902	744	1,646	42%
*Rattus n.*	5,341	461	423	884	49%
*Mus m.*	23,120	767	776	1,543	38%
*Homo s.*	28,133	1,047	1,042	2,089	34%

Number of full length cDNA sequences in each organism is shown. Last column indicates the percent of matched contig sequences that extend to within 5 aa of the start codon of the matching full length cDNA.

**Figure 6:**
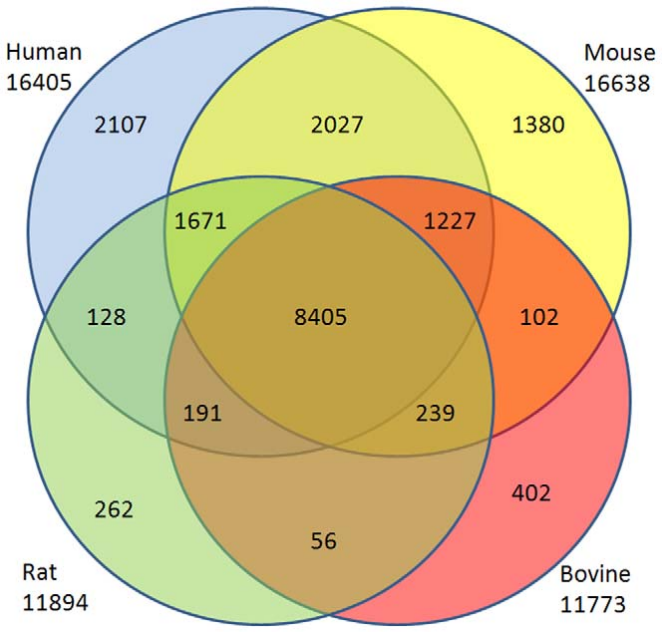
Shared Genes: Comparison of genes found (and then matched in HomoloGene) in human, mouse, rat, and bovine. Regions not shown in the Venn diagram are genes shared by human and bovine only (403 genes) and mouse and rat only (536 genes).

**Figure 7:**
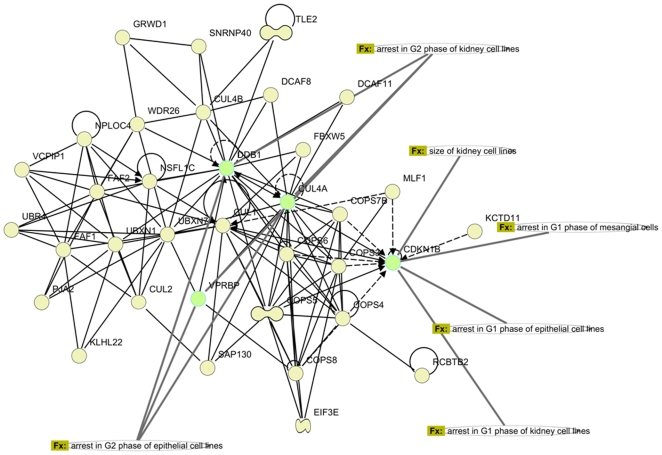
Gene Interaction Network: Most significant network identified by IPA using 8,405 genes found in camel ESTs shared by human, mouse, rat, and bovine. Molecules involved in two most highly associated functions in the network (“hair and skin development and function” and “renal and urological system development” are shown in light green with related functional annotation.

**Figure 8:**
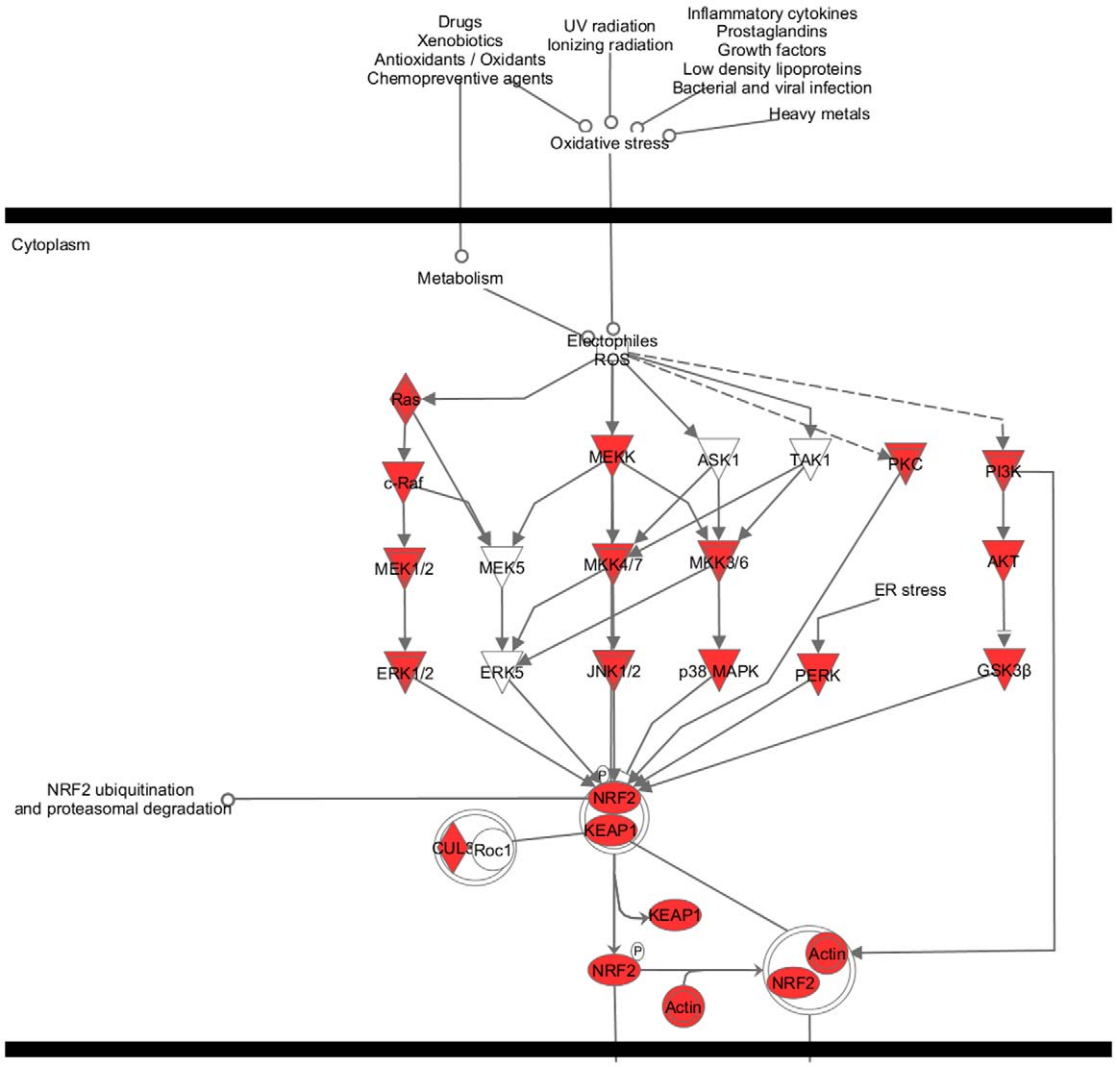
Canonical Pathway: Most significantly associated pathway (NRF-2 mediated oxidative stress response pathway) by the data set of 8,405 genes found in camel ESTs shared by human, mouse, rat, and bovine using IPA. Molecules that exist in the data set are shown in red.

EST sequences have mapped to 22,980; 22,212; 14,991; and 14,766 genes in human, mouse, rat, and bovine, respectively. We first matched these genes to HomoloGene Group IDs, resulting in 16,405; 16,638; 11,834; and 11,773 matches in human, mouse, rat, and bovine, respectively. When these lists were compared, we found 8,405 genes shared by all four organisms constituting a large portion of genes identified in each organism. Figure 6 shows Venn diagram depicting comparison of genes found (and then matched in HomoloGene) in four organisms. Regions not shown in the Venn diagram are genes shared by human and bovine only (403 genes) and mouse and rat only (536 genes). Complete lists of genes for each region of Venn diagram can be found in CAGBASE. In Table 9, we list top 50 genes shared by all four organisms based on their collective frequency of occurrence. This list varies from the genes shown in Table 3, Table 4, Table 5, and Table 6 as most of the genes shown in Table 3, Table 4, Table 5, and Table 6 are not mapped by HomoloGene database. IPA analysis of shared genes revealed a network with 35 molecules where most significantly associated biological functions were “hair and skin development and function” and “renal and urological system development” (p<10^−5^), This network along with sample molecules participating in aforementioned functions are depicted in Figure 7. In Figure 8, we show part of the top canonical pathway, NRF-2 mediated oxidative stress response (p<10^−10^), which is most significantly associated with shared genes based on IPA.

**Table 9:** Top 50 genes in camel that are shared by human, mouse, rat, and bovine.

GeneID	Gene_Symbol	Gene Name
75314	HNRPDL	heterogeneous nuclear ribonucleoprotein D-like
22410	HNRNPD	heterogeneous nuclear ribonucleoprotein D (AU-rich element RNA binding protein 1)
55558	ANXA6	annexin A6
1669	ITIH3	inter-alpha (globulin) inhibitor H3
1667	ITIH1	inter-alpha (globulin) inhibitor H1
68258	MYH11	myosin, heavy chain 11, smooth muscle
23165	HNRNPH2	heterogeneous nuclear ribonucleoprotein H2 (H′)
113602	ZNF184	zinc finger protein 184
68164	ANXA4	annexin A4
20626	PTPRS	protein tyrosine phosphatase, receptor type, S
74950	HNRNPAB	heterogeneous nuclear ribonucleoprotein A/B
21416	KLHL2	kelch-like 2, Mayven (Drosophila)
20716	USP4	ubiquitin specific peptidase 4 (proto-oncogene)
73874	COL1A1	collagen, type I, alpha 1
23741	HMCN1	hemicentin 1
55857	ACTN4	actinin, alpha 4
51861	ZNF569	zinc finger protein 569
79542	KLHL3	kelch-like 3 (Drosophila)
22759	ANXA11	annexin A11
113709	CYP2C19	cytochrome P450, family 2, subfamily C, polypeptide 19
55941	MYH10	myosin, heavy chain 10, non-muscle
13066	USP32	ubiquitin specific peptidase 32
65318	ZNF629	zinc finger protein 629
45	C2	complement component 2
55553	ACTN1	actinin, alpha 1
8306	ZNF180	zinc finger protein 180
862	ACTN3	actinin, alpha 3
22695	ANGPTL2	angiopoietin-like 2
20623	PTPRF	protein tyrosine phosphatase, receptor type, F
74294	HSPA1B	heat shock 70kDa protein 1B
36149	ANXA7	annexin A7
21268	ZNF192	zinc finger protein 192
74536	PCBP2	poly(rC) binding protein 2
48343	PRKCE	protein kinase C, epsilon
20482	GRSF1	G-rich RNA sequence binding factor 1
55437	FGFR3	fibroblast growth factor receptor 3 (achondroplasia, thanatophoric dwarfism)
11012	HNRNPH3	heterogeneous nuclear ribonucleoprotein H3 (2H9)
5169	VIL1	villin 1
1670	ITIH4	inter-alpha (globulin) inhibitor H4 (plasma Kallikrein-sensitive glycoprotein)
68144	UGT2B4	UDP glucuronosyltransferase 2 family, polypeptide B4
75207	CA13	carbonic anhydrase XIII
55433	COL3A1	collagen, type III, alpha 1 (Ehlers-Danlos syndrome type IV, autosomal dominant)
21165	NR1H3	nuclear receptor subfamily 1, group H, member 3
47995	AP1G1	adaptor-related protein complex 1, gamma 1 subunit
38170	FBLN5	fibulin 5
22878	LPHN3	latrophilin 3
20916	CD151	CD151 molecule (Raph blood group)
56926	PCBP4	poly(rC) binding protein 4
1312	BTN1A1	butyrophilin, subfamily 1, member A1
14805	KLHL18	kelch-like 18 (Drosophila)

## Discussion

We have performed large scale EST sequencing of *Camelus Dromedarius* as the first phase of whole genome sequencing of the organism. This work is the first large scale sequencing for camel and has provided over 4,500 potentially novel or fast evolving camel gene sequences that do not carry any homology to other available genomes. Our goal in this work was to generate a comprehensive list of coding sequences found in camel genome irrespective of any specific phenotype. For this purpose, we pooled samples from different tissue, age, and breed. We believe inclusion of a more diverse sampling would yield a better coverage of camel transcriptome but current sample set coupled with our cDNA library normalization strategy should suffice for an initial phase study identifying camel genes. Our results based on read quality, homology, ORF, GO based functional analysis, full length cDNA, and comparative genomic analysis suggest that we have successfully found genes in camel matched to known coding sequences in other organisms and novel gene sequences with a protein coding capacity.

When putative camel gene sequences were analyzed for ORF length based on their hit status, we observed significantly shorter ORFs in sequences with no hit. These results suggest that ORF length, not the sequence length, is a better indicator of finding transcripts with protein coding capacity and subsequently getting a hit in sequence databases. On the other hand, still more than one third of the sequences that got no hit contained an ORF greater than 300 bp and we believe that sequences that got no hit with a relatively long ORF represent novel genes with protein coding capacity. Our results also showed a higher no hit percentage in singleton sequences. We believe that the reason for this is possibly due to the fact that singletons represent rare genes in camel genome that is not well described in other organisms.

It is common to see up to 20% redundancy in large scale EST projects [28]. Instead of assessing such a redundancy in our data set, as no reference sequence set for camel exists, we took advantage of this behavior by assessing genes that got most hits by camel sequences for nine analyzed sequences (see Table 3, Table 4, Table 5, Table 6 and CAGBASE). There is considerable consensus in the lists, which are dominated by immunoglobulin heavy-chain genes (known to be naturally occurring in camel), genes that belong to major histocompatibility classes, and zinc finger proteins. Regarding the IgG isotypes in camels as compared to other species, many authors reported that the numbers of IgG isotypes in different mammalian species vary considerably, depending on the number of functional genes, ranging from one in rabbit [29], three in cattle [30], four in human [31], mouse [32] and rat [33], five in pig [34] and six in horse [35]. It was speculated that increase in the number of IgG isotypes might be accompanied by an increase in functional diversity [36]. Sequences of distinct cDNAs homologous to zinc finger proteins indicate that alternative splicing events adjoin either coding or non coding exons to the finger preceding box sequences [37]. Genes of histocompatibility classes are found in all vertebrates, though the gene composition and genomic arrangement may vary widely due to gene duplication. It was reported that Xanthine oxidoreductase (XOR) activity of camels was markedly lower than that of human, bovine and goat enzymes obtained under the same conditions [38]. This work suggested that the molybdo-form of camel enzyme is totally under desulpho inactive form. It is possible that camel neonates are equipped with an enzymic system that reactivates XOR in their gut and consequently generates antibacterial reactive oxygen species. There are sporadic instances of unidentified genes that have been highly matched by camel sequences, which warrant further biological investigation.

GO analysis revealed expected categories such as “transcription”, “translation”, and “cell cycle” (see Figure 5), however, particularly interesting in camel, one of the overrepresented categories was “oxidation reduction”. Psychosocial stress generally increases oxidative stress promoting a pro-inflammatory environment potentially through facilitation of NF-kappa B between stress and oxidative cellular activation [39], [40]. Oxidative phosphorylation and generation of reactive oxidative species diminishes the negative effect of stress [41]. In this context, surfacing of oxidation reduction process in camel among highly abundant biological processes found in our camel EST database, agrees with camel being a low tempered species with relatively slow movements [4]. Moreover, this observation agrees with recent findings that camel milk alleviates oxidative stress [42] and common infections in camel is associated with a state of oxidative process [43]. There were 514 camel ESTs matched to human genes that were associated with oxidation reduction category including cytochrome c oxidase related genes (COX family), ubiquinol-cytochrome c reductase binding protein (UQCRB), and genes from proxiredoxin (PRDXx) family. Another GO category revealed to be significant that stand outs from generally expected processes is “sensory perception of smell”, which is known to be extremely good in camel [9]. There were 195 camel ESTs matched to human genes that were associated with perception of smell including a broad coverage of olfactory receptors, dopamine transporter (SLC6A3) and receptor (DRD2) and glutamate receptors (GRMx).

The list of most frequently matched 50 out of 8,405 genes found in camel and shared by human, mouse, rat, and bovine was dominated by globulin inhibitors (ITIH family) and zinc finger proteins (ZNF family; see Table 9). ZNFx play a role in diverse functions including lipid binding [44], which may be relevant in camel as camel uses extensive reservoirs of fat to adapt to its environment. The top network generated by IPA using shared genes was associated with hair and skin development, which is extremely important in camel as its thick coat and fur are among the most prioritized adaptations in camel. This network, depicted in Figure 7, holds damage specific DNA binding protein 1 (DDB1) and cyclin dependent kinase inhibitor 1 (CDKN1B) as its hubs. DDB1, CDKN1B, along with cullin 4A (CUL4A) and Vpr binding protein (VPRB) participate in hair and skin development by functioning in arrest of epithelial cell lines in G1 phase [45] and G2 phase [46]. The other most significantly associated function with this network was renal and urological system development, which are important functionalities in camel as its kidneys and intestines efficiently retain water as part of camel's adaptation [9]. This function was facilitated by control of aforementioned four genes in size of kidney cell lines [47], arrest of kidney cell lines in G1 phase [45] and G2 phase [46], and arrest of mesangial cells in G1 phase [48]. In Figure 7, associated functions and genes (in light green) are shown along with complete interaction of all 35 molecules participating in this network. In IPA analysis of 8,405 genes, most significantly associated pathway with the data set came out to be “NRF-2 mediated oxidative stress response pathway”, validating the results of independent GO analysis (see Figure 8). This pathway, which is mostly regulated by mitogen activated protein kinases –all found in the camel ESTs- is anchored by NRF-2. NRF-2 is a key regulator of antioxidant response [49], deficiency of which results in increased quantities of reactive oxygen species [50]. In Figure 8, we show a part of this pathway where genes in the input data set are indicated in red.

In order to share our findings with the scientific community, we developed a web portal hosting the EST database and analysis results at http://camel.kacst.edu.sa. The web portal is made public domain with possibility to add sequences to the existing database. A number of tools are also made available such as the ability to BLAST against our EST sequences. The database has been named CAGBASE, after *Camel Genome Database*. Through this web portal, we present a relational database showing all camel sequences along with corresponding genes and GO terms found for different species. The web portal accepts queries with GeneID, gene symbol, gene name, or GO ID to find camel sequences associated with any one of the query terms. We hope that this web portal provides a home base for genetic studies regarding *Camelus Dromedarius*.
